# The first record of a fly of the family Milichiidae (Diptera) interacting with an ant of the genus *Polyrhachis* Smith, 1857 (Hymenoptera: Formicidae)

**DOI:** 10.3897/BDJ.2.e4168

**Published:** 2014-11-14

**Authors:** Kalsum M Yusah, Tom Maurice Fayle

**Affiliations:** † Institute for Tropical Biology and Conservation, Universiti Malaysia Sabah, 88400 Kota Kinabalu, Sabah, Malaysia, Kota Kinabalu, Malaysia; ‡ Forest Ecology and Conservation Group, Imperial College London, Silwood Park Campus, Buckhurst Road, Ascot, Berkshire, SL5 7PY, London, United Kingdom; § Faculty of Science, University of South Bohemia and Institute of Entomology, Biology Centre of Czech Academy of Sciences, České Budějovice, Czech Republic

**Keywords:** Formicidae, *Polyrhachis illaudata*, *
Myrma
*, Milichiidae, *
Milichia
*, kleptoparasitism, rain forest

## Abstract

Flies in the family Milichiidae are often myrmecophilic. We document the first record of a fly from this family interacting with an ant of the genus *Polyrhachis*. In lowland riparian rainforest in Sabah, Malaysia, we observed a female of the genus *Milichia* following an ant of the species of *P. illaudata*, and repeatedly attempting to make close contact. Our observation suggests that the dipteran may have been attempting to feed kleptoparasitically from the *Polyrhachis* worker, since members of this ant genus often feed on liquid carbohydrate-rich food resources. This is the first time an interaction has been observed between a fly of this family and an ant of this widespread old world tropical genus.

## Introduction

Milichid flies often interact with ants, with either adults feeding kleptoparasitically from foraging ant workers, or larvae feeding on detritus in the nest (Brake 1999, Wild and Brake 2009, Moser and Neff 1971). However, due to the difficulties in observing these interactions, the full range of ant and fly taxa over which this interaction occurs is not clear. Here we present an observation of an interaction between a milichiid and a genus of ant not yet known to be targeted by this fly family.

## Materials and methods

Field observation and collection was conducted during a field course organised by the Naturalis Biodiversity Center at Danau Girang Field Centre on the lower Kinabatangan river, Sabah, Malaysia. On March 4^th^ 2014, in an area of regularly inundated riparian forest 100m from the river (5.4115, 118.0395) close to the field centre, we observed an ant of the genus *Polyrhachis* traversing the top of a plastic sheet c. 50 cm in height, which was being used as a vertical barrier to trap amphibians on the forest floor during the field course. The single *Polyrhachis* worker was being followed closely (c. 3 cm above and behind) by a hovering dipteran, which frequently attempted to make closer contact with the ant, in particular when the ant stopped moving. The ant was not behaving normally, but appeared to be attempting evasion of the dipteran. We observed this for approximately thirty seconds before collecting both ant and dipteran into a plastic container. The dipteran was initially not disturbed by its collection, and continued for some time with attempts to make close contact with the ant. We were unable to directly observe the outcome of these attempts (either kleptoparasitism or oviposition). Both insects were then preserved in 95% ethanol and point mounted for identification. To identify the ant we first used a key to the ant genera of Borneo (Fayle et al. 2014). We then used a key to subgenus (Dorow 1995) and finally compared the specimen with online images at www.antbase.net and with the BORNEENSIS collection at the Institute for Tropical Biology and Conservation, Universiti Malaysia Sabah (note that there is no published key to the Bornean species for this subgenus). To identify the fly we used the key to genera at http://milichiidae.info/content/key-genera-milichiidae, based on that of Brake (2000). The specimens were imaged using a Leica M165 C stereo microscope and camera. Both specimens were then deposited in the BORNEENSIS collection; accessions numbers HYM 0003736 and DIP 00713 for the ant and fly respectively. Since we were unable to morphologically identify the fly, we removed a leg from the specimen and stored it in 96% ethanol for future DNA-based identification.

## Taxon treatments

### Milichia

Meigen, 1830

#### Materials

- **Type status:** Other material. **Occurrence:** individualCount: 1; sex: female; lifeStage: adult; behavior: following an ant; preparations: whole animal point mounted; disposition: In collection; otherCatalogNumbers: DIP 00713; **Taxon:** scientificName: Milichia; parentNameUsage: Milichiinae; originalNameUsage: *Milichia* Meigen 1830; **Location:** higherGeography: Asia; Malaysia; Sabah; Kinabatangan River Floodplain; continent: Asia; country: Malaysia; stateProvince: Sabah; locality: Kinabatangan River Floodplain; verbatimLocality: Kinabatangan River Floodplain ~10km downstream from Lahad Datu - Sandakan road crossing; verbatimElevation: 30 m; verbatimCoordinates: 5.4115, 118.0395; verbatimLatitude: 5.4115; verbatimLongitude: 118.0395; verbatimCoordinateSystem: decimal degrees; decimalLatitude: 5.4115; decimalLongitude: 118.0395; **Identification:** identifiedBy: Irina Brake; dateIdentified: 13-09-2014; identificationReferences: Brake (2000); identificationRemarks: Not possible to assign *Milichia* females to species.; **Event:** samplingProtocol: Manual collection into plastic pot.; samplingEffort: Single collection event.; eventDate: 2014-03-04; habitat: Riparian lowland rain forest.

### Polyrhachis (Myrma) illaudata

Walker, 1859

#### Materials

- **Type status:** Other material. **Occurrence:** individualCount: 1; sex: female; lifeStage: adult; behavior: foraging; preparations: whole animal point mounted; disposition: in collection; otherCatalogNumbers: HYM 0003736; **Taxon:** scientificName: Polyrhachis (Myrma) illaudata Walker 1859; parentNameUsage: Formicinae; **Location:** higherGeography: Asia; Malaysia; Sabah; Kinabatangan River Floodplain; continent: Asia; country: Malaysia; stateProvince: Sabah; locality: Kinabatangan River Floodplain; verbatimLocality: Kinabatangan River Floodplain ~10km downstream from Lahad Datu - Sandakan road crossing; verbatimElevation: 30 m; verbatimCoordinates: 5.4115, 118.0395; verbatimLatitude: 5.4115; verbatimLongitude: 118.0395; verbatimCoordinateSystem: decimal degrees; decimalLatitude: 5.4115; decimalLongitude: 118.0395; **Identification:** identifiedBy: Tom M. Fayle; dateIdentified: 2014-06-20; identificationReferences: www.antbase.net; The BORNEENSIS collection at the Institute for Tropical Biology, Universiti Malaysia Sabah; Dorow 1995.; **Event:** samplingProtocol: Manual collection into plastic pot.; samplingEffort: Single sampling occasion.; eventDate: 2014-03-04

## Analysis

The dipteran was identified as a female of the genus *Milichia* (Fig. 1), in the family Milichiidae. It is not possible to identify females of this genus to species level (I. Brake, pers. comm.). The ant was identified as belonging to the species *Polyrhachis illaudata* (Fig. 2) in the subgenus *Myrma*. Similar species include *P. obesior*, which differs in the shape of its petiole (ridge between central pair of spines protrudes anteriorly, central spines more greatly divergent than in *P. illaudata*, in which spines curve inwards, becoming almost parallel at tips), and *P. beccarii*, which is morphologically similar, but much larger than *P. illaudata*.

## Discussion

To our knowledge, this is the first record of a fly in the family Milichiidae being associated with an ant in the genus *Polyrhachis*. The only other record of an ant in the tribe Camponotini being associated with this family of flies is that of *Camponotus acvapimensis*, with a fly of the species *Milichia savannaticola* being found with the ant, although it was not clear whether the two species were interacting (Deeming 1981). However, there are other records of interactions between milichiids and ants in the subfamily Formicinae including *Lasius* (Donisthorpe 1927) and *Formica* (Verrall 1894). Although we were unable to observe the interaction directly, we suggest that this *Milichia* female was attempting to feed kleptoparasitically from the *P. illaudata* ant, since many other species of diptera in this family are known to conduct such attacks, either feeding on regurgitated liquid food or through licking anal secretions (Brake 1999). *Polyrhachis* ants, and indeed other genera in the Camponotini, are known to feed extensively on liquid carbohydrate-rich food sources at extrafloral and floral nectaries, and also to attend homopterans (Blüthgen et al. 2003, Davidson et al. 2003), and hence might be particularly vulnerable to this kind of kelptoparasitism. The other common kind of myrmecophilic interaction between diptera in this family and ants is when milichiid larvae are found in ant nests feeding on detritus (Moser and Neff 1971). However, females usually enter the ant nest itself to lay their eggs in these cases (Moser and Neff 1971), and so it seems unlikely that the female we observed would be attempting oviposition on the ant, since this would be a rather risky tactic if parasitism was not to be the final outcome. We suggest that the interaction between these two species be further studied, either in the field or in the laboratory, to confirm its kleptoparasitic nature.

## Supplementary Material

XML Treatment for Milichia

XML Treatment for Polyrhachis (Myrma) illaudata

## Figures and Tables

**Figure 1a.**
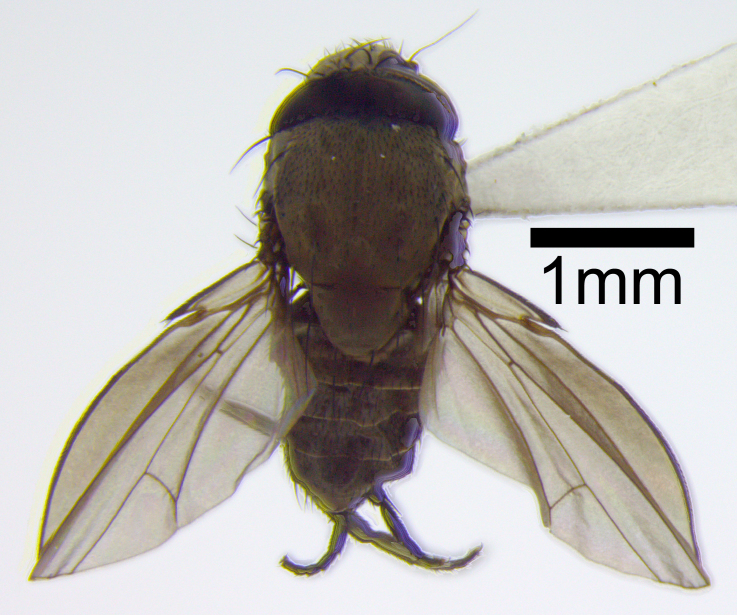
Dorsal view of the *Milichia* sp. female.

**Figure 1b.**
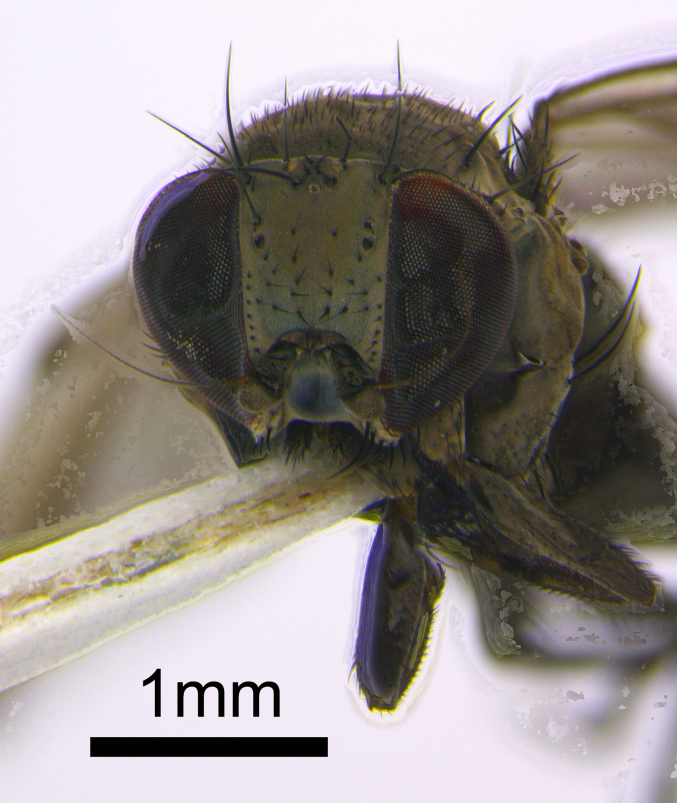
Frontal view of the *Milichia* sp. female.

**Figure 1c.**
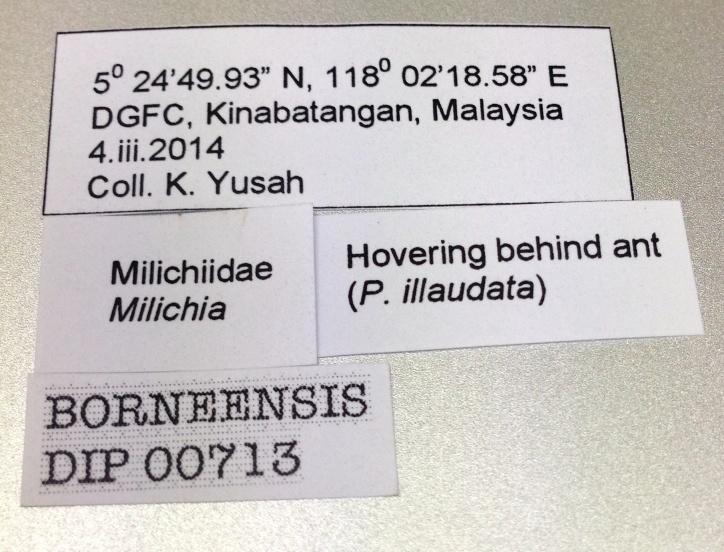
Label for *Milichia* sp. female specimen.

**Figure 2a.**
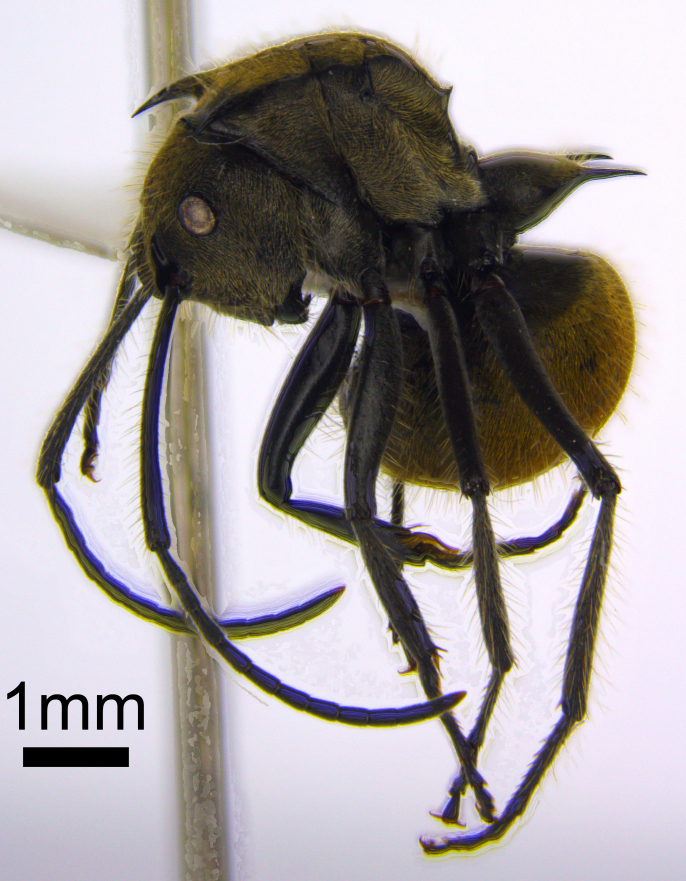
Lateral view of *Polyrhachis illaudata*.

**Figure 2b.**
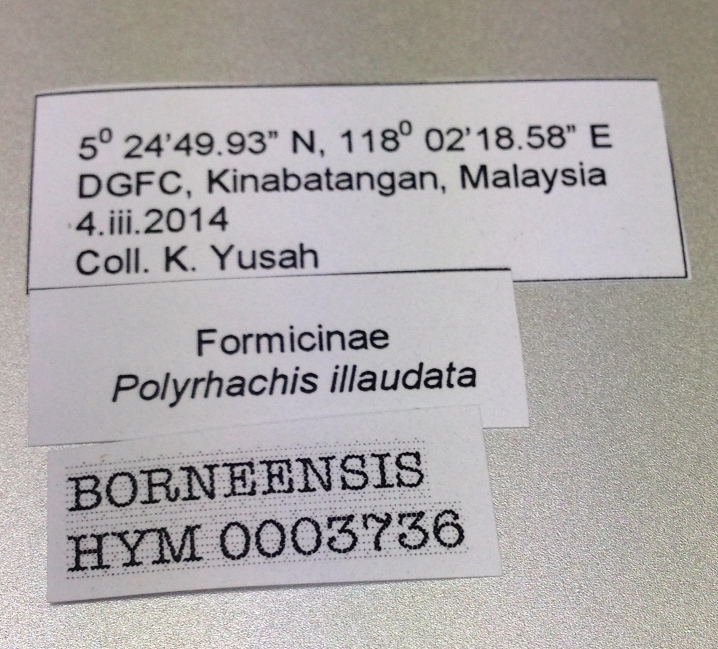
Label for *Polyrhachis illaudata* specimen.
